# Catheter-directed mechanical aspiration thrombectomy in a real-world pulmonary embolism population: a multicenter registry

**DOI:** 10.1093/ehjacc/zuad066

**Published:** 2023-06-15

**Authors:** Sylwia Sławek-Szmyt, Jakub Stępniewski, Marcin Kurzyna, Wiktor Kuliczkowski, Stanisław Jankiewicz, Grzegorz Kopeć, Szymon Darocha, Ewa Mroczek, Arkadiusz Pietrasik, Marek Grygier, Maciej Lesiak, Aleksander Araszkiewicz

**Affiliations:** Department of Cardiology, Poznan University of Medical Sciences, Długa 1/2 Street, 61-848 Poznan, Poland; Pulmonary Circulation Centre, Department of Cardiac and Vascular Disease, Jagiellonian University Medical College, John Paul II Hospital in Krakow, Prądnicka 80 Street, 31-202 Krakow, Poland; Department of Medical Education, Jagiellonian University Medical College, Medyczna 7 Street, 30-688 Krakow, Poland; Department of Pulmonary Circulation, Thromboembolic Diseases and Cardiology European Health Centre Otwock, Medical Centre for Postgraduate Education, Borowa 14/18 Street, 05-400 Otwock, Poland; Department of Cardiology, Wroclaw Medical University, Borowska 213 Street, 50-556 Wroclaw, Poland; Department of Cardiology, Poznan University of Medical Sciences, Długa 1/2 Street, 61-848 Poznan, Poland; Pulmonary Circulation Centre, Department of Cardiac and Vascular Disease, Jagiellonian University Medical College, John Paul II Hospital in Krakow, Prądnicka 80 Street, 31-202 Krakow, Poland; Department of Pulmonary Circulation, Thromboembolic Diseases and Cardiology European Health Centre Otwock, Medical Centre for Postgraduate Education, Borowa 14/18 Street, 05-400 Otwock, Poland; Department of Cardiology, Wroclaw Medical University, Borowska 213 Street, 50-556 Wroclaw, Poland; Department and Faculty of Cardiology, Medical University of Warsaw, Banacha 1A Street, 02-097 Warsaw, Poland; Department of Cardiology, Poznan University of Medical Sciences, Długa 1/2 Street, 61-848 Poznan, Poland; Department of Cardiology, Poznan University of Medical Sciences, Długa 1/2 Street, 61-848 Poznan, Poland; Department of Cardiology, Poznan University of Medical Sciences, Długa 1/2 Street, 61-848 Poznan, Poland

**Keywords:** Catheter-directed mechanical aspiration thrombectomy, High-risk, Intermediate-high risk, Mortality, Pulmonary embolism, Safety

## Abstract

**Aims:**

High- (HR) and intermediate-high risk (IHR) pulmonary embolisms (PEs) are related to high early mortality and long-term sequelae. We aimed to describe clinical outcomes and adverse events in IHR and HR pulmonary embolism (PE) treated with catheter-directed mechanical thrombectomy (CDMT) in a real-world population.

**Methods and Results:**

This study is a multicenter, prospective registry enrolling 110 PE patients treated with CDMT between 2019 and 2022. The CDMT was performed using the 8F Indigo (Penumbra, Alameda, CA, USA) system bilaterally in pulmonary arteries (PAs). The primary safety endpoints included device or PE-related death during the 48-h after CDMT, procedure-related major bleeding, or other major adverse events. Secondary safety outcomes were all-cause mortality during hospitalization or the follow-up. The primary efficacy outcomes were the reduction of PA pressures and change in the right-to-left ventricular (RV/L) ratio assessed in the imaging 24–48 h after the CDMT.

71.8% of patients had IHR PE and 28.2% HR PE. 11.8% of patients had a failure and 34.5% had contraindications to thrombolysis, and 2.7% had polytrauma. There was 0.9% intraprocedural death related to RV failure and 5.5% deaths within the first 48 h. CDMT was complicated by major bleeding in 1.8%, pulmonary artery injury in 1.8%, and ischaemic stroke in 0.9%. Immediate haemodynamic improvements included a 10.4 ± 7.8 mmHg (19.7%) drop in systolic PAP (*P* < 0.0001), a 6.1 ± 4.2 mmHg (18.8%) drop in mean PAP, and 0.48 ± 0.4 (36%) drop in RV/LV ratio (*P* < 0.0001).

**Conclusion:**

These observational findings suggest that CDMT may improve hemodynamics with an acceptable safety profile in patients with IHR and HR PE.

## Graphical Abstract

**Graphical Abstract:**
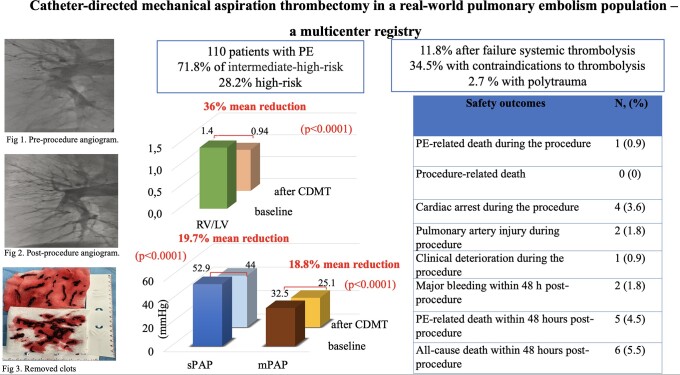

## Introduction

Over the last decade, the role of endovascular therapies in the management of acute pulmonary embolism (PE) has rapidly evolved.^1–6^ Catheter-directed thrombolysis and catheter-directed mechanical thrombectomy (CDMT) techniques have been developed to counter the bleeding risk associated with systemic thrombolysis (ST).^3,7–9^ While CDMT for the treatment of intermediate-high risk (IHR) and HR PE is becoming increasingly implemented, data supporting its safety, and efficacy have been limited.^8,10–12^ The paucity of clinical outcomes research in advanced stages of PE (IHR and HR), especially for endovascular therapies limits its implementation by the current recommendations’ guidelines.^2,3,9,13^ CDMT uses a vacuum source providing aspiration through a catheter to achieve a percutaneous thrombectomy.^8^ The Indigo system (Penumbra, Alameda, CA, USA) is an 8F CDMT device designed to remove clots from PAs without using thrombolysis. The effectiveness of the Indigo system in the PE treatment was demonstrated in the EXTRACT-PE study, but the patient cohort was restricted to intermediate-high-risk (submassive) PE patients.^8^ This report aims to evaluate outcomes of the Indigo aspiration system, including immediate changes in hemodynamics, acute safety, and effectiveness, and longer-term clinical outcomes during 90-day follow-up.

## Methods

### Study design

The study is a prospective multicenter registry that enrolled 110 patients at four centres in Poland between January 2019 and August 2022. The study protocol was in accordance with the Declaration of Helsinki and was approved by the coordinating centre’s institutional bioethics committee (Poznan University of Medical Sciences Bioethics Committee; approval number 271/2021). Data for this study were obtained from the larger prospective, multicenter registry of patients treated by Polish PERTs; the Polish Multicenter PE Response Teams Outcomes Registry registered in ClinicalTrials.gov: NCT04879069. All patients provided informed consent (if they were unconscious, close relatives approved the treatment).

Inclusion criteria were patients older than 18 years old with symptoms of acute PE for less than 14 days who were categorized as IHR, or HR in accordance with the guidelines of the European Society of Cardiology (ESC), and qualified for CDMT with the Indigo System per the local PE response team (PERT) discretion.^2,14–16^ Enrolled patients had proximal clots located in at the minimum one main or lobar pulmonary artery (PA) confirmed by computed tomography pulmonary angiography (CTPA). Patients with IHR PE were enrolled if they demonstrated at least one of the listed signs of clinically severe PE longer than 24 h despite proper parenteral anticoagulation: heart rate (HR) ≥ 100 bpm, systolic blood pressure (SBP) 90–100 mmHg, arterial blood saturation (SaO_2_) ≤ 90% on room air and were considered to be at increased risk of bleeding complications when treated with full-dose ST. Patients with IHR PE and haemodynamic deterioration (sudden occurrence of one or more of the abovementioned signs of severe PE) were also included. HR PE patients with SBP < 90 mmHg or requiring catecholamine support to keep SBP > 90 mmHg, or after cardiac arrest, were enrolled if they had absolute contraindications to ST, or previously administered thrombolysis failed.

Exclusion criteria were pregnancy, refusal to sign the informed consent form, presence of intracardiac thrombus, history of severe or chronic pulmonary hypertension, known serious, and uncontrolled sensitivity to radiographic agents, a life expectancy of less than a month due to co-morbidities (as determined by the physician), severe thrombocytopenia (platelets count below 20 000/µL), or active major bleeding according to the Bleeding Academic Research Consortium (BARC).^17^ Follow-up evaluations were performed at 24–48 h, 30 days (±7 days), and 90 days (±21 days). Demographic, clinical, laboratory, and mandatory imaging studies (echocardiographic and CTPA) data were collected in all patients. The analysis of the imaging studies was performed by two independent reviewers from the coordinating centre, a cardiologist with an experience in echocardiography and a radiologist with experience in interpreting the CTPA of the PAs. All participating centres supported the study with internal funding from statutory funds.

### Catheter-directed mechanical aspiration thrombectomy

The Indigo aspiration System comprises the 8F Indigo aspiration catheter and associated tubing, vacuum pump, and separator wire used for mechanical thrombus fragmentation.^8^ CDMT procedures were mostly performed via the common femoral vein access. To evaluate hemodynamics 7F Swan-Ganz catheter was used, and cardiac output and pulmonary vascular resistance were assessed with the Fick method. Then, a diagnostic pulmonary angiogram via a 6F pigtail catheter was performed to indicate detailed thrombi burden. Thrombi load was assessed according to the Miller Index.^18,19^

**Figure 1:**
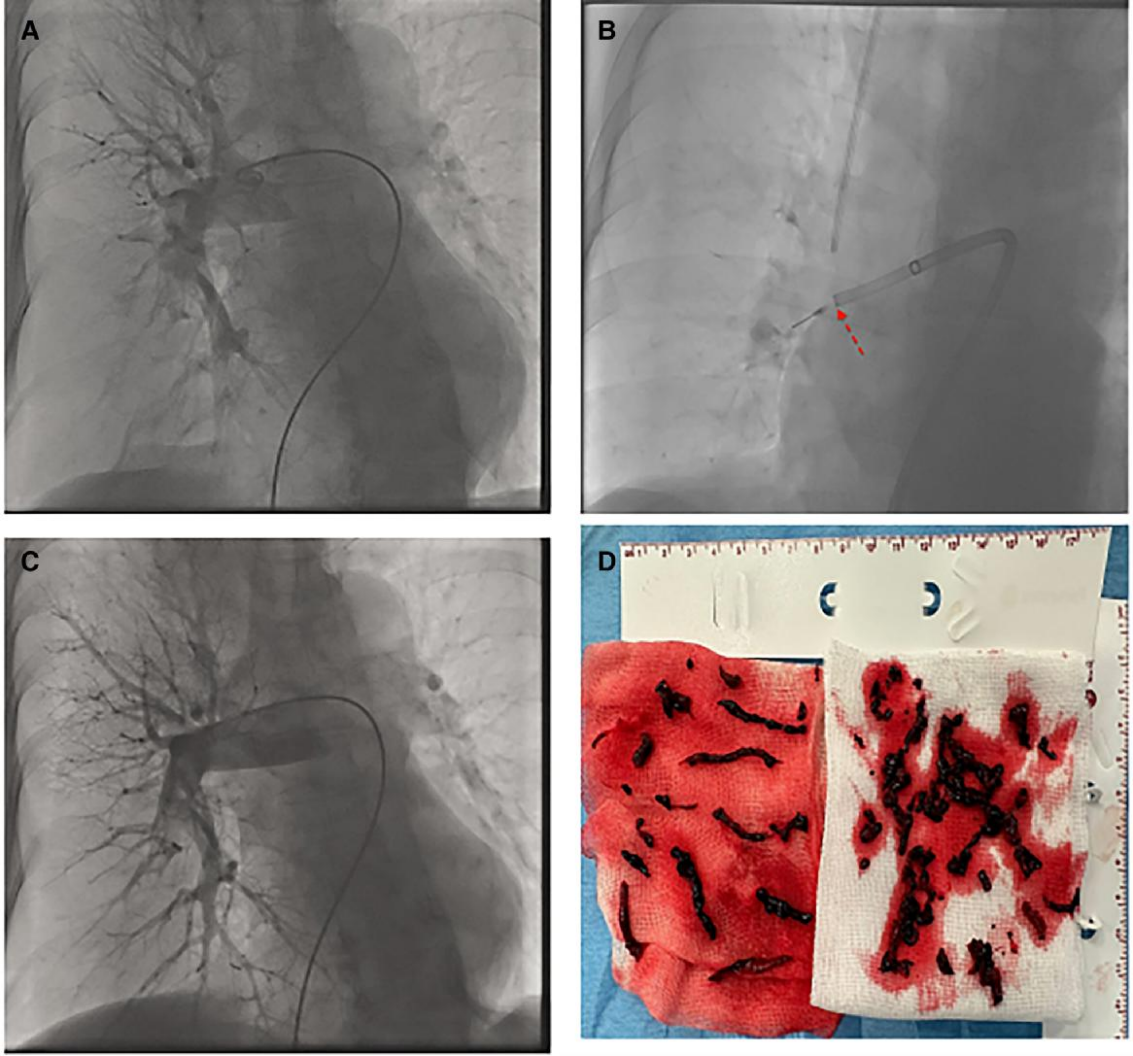
Catheter-directed mechanical aspiration thrombectomy procedure. (*A*) Pulmonary angiogram showing clots in right main and lobar pulmonary arteries with total occlusion of intermediate and lower lobe arteries. (*B*) Catheter-directed mechanical aspiration thrombectomy with the separator wire used in the right lower lobe artery. (*C*) Pulmonary angiogram showing clots removal from lower in the right lower lobe artery. (*D*) Image of clots removed from pulmonary arteries during the procedure.

The 8F Indigo catheter was introduced over a 0.035-in guidewire and deployed proximal to the thrombus in the lobar artery of the right or left PA, and sustained aspiration was initiated to remove the clot. Then separator wire was repeatedly passed through the thrombus to break it up and facilitate suction. The above manoeuvres could be repeated in multiple vessels. A final angiogram and haemodynamic assessment, including PA pressures (PAPs) were performed directly after CDMT and recorded (see *Figure 1*). Procedural anticoagulation was provided with therapeutic doses of unfractionated heparin (UFH) under activated clotting time control (therapeutic range: 200–300 s.). Investigators determined when to terminate the intervention based on carefully evaluating the patient’s haemodynamic status, residual thrombus burden, and the total amount of aspirated blood (should not exceed 300 mL). After CDMT UFH or low-molecular-weight heparin (LMWH) in weight-adjusted dose was continued for 24–48 h depending on the patient’s clinical status, and the type of anticoagulation drug administered to each patient at discharge was at the investigator’s discretion.

**Figure 2:**
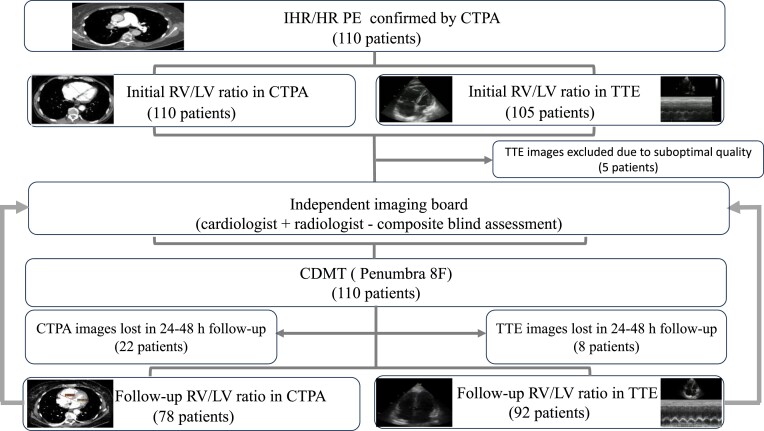
Flowchart of imaging studies analysis.

### Outcomes

The primary safety endpoints included device or PE-related death during the 48-h after CDMT, procedure-related major bleeding (BARC type 3a or greater), or other procedure-related major adverse events.^17^ Secondary safety outcomes were all-cause mortality during the index hospitalization or follow-up period. CDMT or device-related adverse events were specified as PA injury, cardiac injury, cardiac tamponade, sustained ventricular tachyarrhythmias, clinical deterioration defined by haemodynamic, or respiratory meeting specific thresholds, access-site pseudoaneurysm, arteriovenous fistula, peripheral ischaemia, or nerve injury. Clinical safety outcomes were assessed by trained independent physician reviewers not involved in performing the procedures.

The reduction of PAPs (systolic PAP and mean PAP) and the reduction in the right RV-to-left ventricular ratio (RV/LV ratio) assessed in the CTPA (using the reformatted four-chamber view)^20^ and/or echocardiography (using four-chamber view), 24–48 h after the CDMT were primary efficacy outcomes. Secondary efficacy measures were the improvement in RV strain [tricuspid annular plane excursion (TAPSE)] assessed in the echocardiography 24–48 h after the CDMT, reduction of heart rate (HR), oxygen demand, and change in blood pressure as well as symptoms relief. All CTPA studies were of good quality for analysis, but five initial transthoracic echocardiography (TTE) scans had suboptimal quality and were excluded from the analysis. Details regarding imaging acquisition and analysis are displayed in *Figure 2*.

### Statistical analysis

Patients’ characteristics are presented as absolute and percentage frequencies for categorical variables and mean with standard deviation (SD) for continuous variables with normal distribution. The normality distribution was assessed with the Shapiro–Wilk test. Variables with a non-normal distribution are presented as the median and interquartile range (IQR). The differences between the parameters at baseline, after CDMT, and follow-up were analysed using the paired Student’s *t*-test or Wilcoxon signed-rank test where appropriate. McNemar’s test was applied to compare categorical variables using available pairwise values. The associations between baseline characteristics and RV/LV ratio reduction 48 h post-procedure were evaluated using multiple linear regression models. Statistical analysis was performed using MedCalc Software Ltd. version 20.215 (Ostend, Belgium).

## Results

### Baseline patients’ characteristics

The mean age was 57.3 ± 13.5 years, and 38.2% of patients were female. According to current ESC guidelines, 28.2% were categorized as HR PE, and 71.8% as IHR PE. The mean Pulmonary Embolism Severity Index (PESI) score among IHR patients was 120.3 ± 32.4 points. Notably, 11.8% of patients underwent unsuccessful ST before CDMT, 4.5% deteriorated on parenteral anticoagulation, and 34.5% were assessed to have absolute contraindications to thrombolysis according to local PERT. The baseline composite RV/LV ratio was 1.4 ± 0.3. All patients with initial laboratory work-up (100 of 110) had elevated highly sensitive (hs) Troponin I and N-terminal pro-brain natriuretic peptide (NT-proBNP) levels. The mean baseline hs Troponin I level was 2.4 ± 7.3 ng/mL (normal value <0.05 ng/mL), and the mean baseline NT-proBNP level was 5609.7 ± 5805.3 pg/mL (normal value <125 pg/mL). Concomitant deep vein thrombosis was present in 60.9% of patients. Moreover, 14.5% of patients underwent surgery within 2 weeks before PE diagnosis and 2.7% were polytrauma patients. Details are presented in *Table 1*.

**Table 1:** Baseline patients’ demographics and clinical characteristics

Variables	Baseline
*Demographics*	
Age, years	57.3 ± 13.5
Female/male sex	42 (38.2)/68 (61.8)
BMI, kg/m^2^	29.0 ± 5.0
*Co-morbidities*	
Arterial hypertension	40 (36.4)
Congestive heart failure	3 (2.7)
Diabetes mellitus	11 (10.0)
Concomitant deep vein thrombosis	67 (60.9)
Coronary artery disease	4 (3.6)
Chronic obstructive pulmonary disease	10 (9.1)
COVID-19	1 (0.9)
Chronic kidney disease	10 (9.1)
*Risk factors for PE*	
Obesity, BMI ≥ 30 kg/m^2^	35 (31.8)
Malignancy	19 (17.3)
Surgery within last 2 weeks	16 (14.5)
Polytrauma	3 (2.7)
Immobilization	14 (12.7)
Thrombophilia	20 (18.2)
History of PE	25 (22.7)
Oral contraceptive	7 (6.4)
*PE clinical presentation*	
Syncope	13 (11.8)
Dyspnea at rest	100 (90.9)
Chest pain	5 (4.5)
Symptoms duration, days	3 ± 2.5
*PE severity*	
Intermediate-high risk	79 (71.8)
High risk	31 (28.2)
PESI score	120.3 ± 32.4
PESI class	
PESI class	
I-II	3 (2.7)
III	6 (5.5)
IV	41 (37.3)
V	29 (26.4)
Heart rate, bpm	110.7 ± 16.8
Systolic blood pressure, mmHg	115 ± 22.2
Diastolic blood pressure, mmHg	74.9 ± 15.4
Respiratory rate, pm	29.5 ± 5.4
Oxygen supplementation (to keep SaO_2_ > 90%)	80 (72.7)
Arterial oxyhaemoglobin saturation, %	90.2 ± 6.0
Arterial pO_2_, mmHg	66.9 ± 21.2
FiO_2_,	0.6 ± 0.3
Oxygenation index (pO_2_/FiO_2_)	122.5 ± 113.5
*Biomarkers*	
hs Troponin I level, ng/mL (normal value <0.05 ng/mL)	2.4 ± 7.3
NT-proBNP level, pg/mL (normal value < 125 pg/mL)	5609.7 ± 5805.3
Lactate level, mmol/L (normal value <2.2 mmo/L)	2.8 ± 2.0
*RV dysfunction*	
RV/LV ratio, mm/mm (CTPA)	1.4 ± 0.3
RV/LV ratio, mm/mm (echo)	1.3 ± 0.3
Composite RV/LV ratio, mm/mm (CTPA/echo)^a^	1.4 ± 0.3
TAPSE, mm	15 ± 4.1
S’ wave, cm/s	11.5 ± 3.3
RVSP, mmHg	51.1 ± 12.6
*Failed therapy prior CDMT*	
ST	13 (11.8)
Anticoagulation (clinical deterioration during therapy)	5 (4.5)
Absolute contraindication to thrombolysis	38 (34.5)

Composite RV/LV ratios using either CTPA or TTE measurements, with CTPA prioritized if both were available.

Abbreviations: BMI, Body mass index; CDMT, catheter-directed mechanical thrombectomy; FiO_2_, oxygen demand; hs, high sensitive; NT-proBNP, N-terminal-pro-B-type natriuretic peptide; pO_2_, oxygen partial pressure; PE, pulmonary embolism; PESI, pulmonary embolism severity index; RV/LV ratio, right ventricular-to-left ventricular ratio; RVSP, right ventricular systolic pressure; ST, systemic thrombolysis; TAPSE, tricuspid annular systolic excursion.

### Procedural characteristics

All patients presented with bilateral PE, and 50.9% of patients had clots mostly located in the main and lobar PAs. Most patients (56.4%) received UFH as initial anticoagulation. In the vast majority of cases (98.2%), the CDMT was performed via common femoral vein access. There were two (1.8%) major access-site haematomas that were treated conservatively but required transfusion of two units of packed red blood cells. The mean total procedure duration time (skin-to-skin) was 64.7 ± 22.0 min, with the mean estimated blood loss per procedure of 296 ± 79.2 mL. In three HR PE patients (2.7%), the CDMT was performed on extracorporeal membrane oxygenation (ECMO) support. An inferior vena cava filter was implanted in four patients (3.6%) due to absolute contraindications to anticoagulation. In one case (0.9%) due to the co-existence of chronic thromboembolic lesions, the CDMT was escalated to open surgical thrombendarterectomy. 10.9% of patients were hospitalized in the intensive care unit (ICU) after the procedure, with a mean ICU stay of 5 ± 4 days. Patients were mostly discharged on direct oral anticoagulants 59% followed by low-molecular-weight-heparins in 18.2% and vitamin K antagonists in 8.2%. Procedural characteristics are summarized in *Table 2*.

**Table 2:** Procedural characteristics and clinical outcomes

Variables	
Time from diagnosis to procedure, days, mean ±SD	1.6 ± 1.4
Initial anticoagulant	
Unfractionated heparin	62 (56.4)
Low-molecular-weight heparin	41 (37.3)
PE location	
Bilateral	110 (100)
Saddle and main arteries	56 (50.9)
Lobar and segmental	54 (49.1)
Segmental	—
Access-site	
Common femoral vein	108 (98.2)
Jugular vein	2 (1.8)
Access-site complications	
Major ^a^	2 (1.8)
Minor	8 (7.3)
Total procedure duration time (min), mean ±SD	64.7 ± 22.0
Amount of blood loss, ml, mean ±SD	296.0 ± 79.2
Periprocedural ECMO use	3 (2.7)
Vena cava filter implantation	4 (3.6)
Conversion to open surgery	1 (0.9)
Length of hospitalization, days, mean ±SD	10 ± 6.8
Length of hospitalization after the procedure, days, mean ±SD	8 ± 5.6
Need for ICU stay	12 (10.9)
Anticoagulation at discharge	65 (59)
Direct oral anticoagulant	9 (8.2)
Vitamin K antagonist	20 (18.2)
Low-molecular-weight heparin None	4 (3.6)

No need for surgical or endovascular treatment.

Abbreviations: See *Table 1*. ECMO, extracorporeal membrane oxygenation; ICU, intensive care unit.

### Clinical and haemodynamic outcomes

Clinical and haemodynamic outcomes are presented in *Table 3*. The mean baseline RV/LV ratio was 1.4 ± 0.3 and the 48-h post-procedure RV/LV ratio was 0.94 ± 0.1 [−0.48 ± 0.4 (36%) mean change, *P* < 0.0001]. Immediately following the procedure, the sPAP decreased from 52.9 ± 16.0 mmHg to 44 ± 13.4 mmHg [−10.4 ± 7.8 mmHg (19.7%) mean change, *P* < 0.0001]. The mPAP also significantly decreased on-table from 32.5 ± 7 mmHg to 25.1 ± 8.5 mmHg [−6.1 ± 4.2 mmHg (18.8%) mean change, *P* < 0.0001]. There was also a significant on-table improvement in mean pulmonary artery pulsatility index (PAPI) from 4.2 ± 2.2 mmHg/mmHg to 7.6 ± 6.6 mmHg/mmHg [+3.0 ± 5.6 mmHg/mmHg (71.4%) mean change, *P* < 0.0001]. Notably, there was a significant oxygen demand (FiO_2_) reduction from 0.6 ± 0.3 to 0.43 ± 0.1 [− 0.27 ± 0.2 (45%) mean change, *P* < 0.0001].

**Table 3:** Changes in hemodynamics and vitals following catheter-directed mechanical thrombectomy

	Initial Mean ± SD	After CDMT Mean ± SD	Mean Change Mean ± SD (%)	*P* value
RV/LV ratio (CTPA)	1.4 ± 0.3	0.94 ± 0.1	−0.48 ± 0.4 (36)	<0.0001
RV/LV ratio (TTE)	1.3 ± 0.3	0.97 ± 0.1	−0.33 ± 0.1 (42.9)	<0.0001
Composite RV/LV (CTPA/TTE) ^a^	1.4 ± 0.3	0.94 ± 0.1	−0.46 ± 0.1 (32.9)	<0.0001
sPAP, mmHg	52.9 ± 16.0	44 ± 13.4	−10.4 ± 7.8 (19.7)	<0.0001
mPAP, mmHg	32.5 ± 7	25.1 ± 8.5	−6.1 ± 4.2 (18.8)	<0.0001
dPAP, mmHg	21.1 ± 5.7	16.8 ± 8.5	−5.9 ± 6.1 (28.0)	<0.0001
mRAP, mmHg	14.1 ± 2.9	11.2 ± 2.4	−3.9 ± 3.5 (27.7)	<0.0001
PAPI, mmHg/mmHg	4.2 ± 2.2	7.6 ± 6.6	+3.0 ± 5.6 (71.4)	<0.0001
CO	4.8 ± 1.3	5.0 ± 1.2	+0.2 ± 0.1 (4.2)	0.0008
TPVR, WU	4.5 ± 2.4	3.3 ± 1.8	−1.2± 0.9 (26.7)	0.003
TAPSE, mm	15 ± 4.1	20.5 ± 4.6	+6.1 ± 4.5 (40.7)	<0.0001
Heart rate, bpm	110.7 ± 16.8	89.5 ± 13.8	−23.9 ± 15.3 (21.6)	<0.0001
SBP, mmHg	115.1 ± 22.3	122.3 ± 13.9	+8.7 ± 20.9 (7.6)	0.07
DBP, mmHg	74.9 ± 15.8	79 ± 10.6	+5.4 ± 15.0 (7.2)	0.15
Arterial pO_2,_ mmHg	66.9 ± 5.5	76.9 ± 2.8	+13.1 ± 17.2 (19.6)	0.0009
FiO_2_	0.6 ± 0.3	0.43 ± 0.1	− 0.27 ± 0.2 (45.0)	<0.0001
hs Troponin I, ng/mL	2.4 ± 0.7	2.1 ± 1.2	−1.6 ± 7.6 (66.7)	<0.0001
NT-proBNP, pg/mL	5609.7 ± 5805.3	3319.7 ± 415.9	− 3277.4 ± 1667.1 (58.4)	<0.0001
Lactate, mmol/L	2.8 ± 0.2	1.9 ± 0.1	−0.9 ± 2.4 (32.1)	<0.0001
Haemoglobin concentration, mmol/L	8.3 ± 0.1	7.7 ± 0.2	−0.8 ± 1.5 (9.6)	<0.0001

Composite RV/LV ratios using either CTPA or TTE measurements, with CTPA prioritized if both were available.

Abbreviations: See *Table 1*. CO, cardiac output; DBP, diastolic blood pressure; dPAP, diastolic pulmonary arterial pressure; mPAP, mean pulmonary arterial pressure; mRAP, mean atrial pressure; PAPI, pulmonary artery pulsatility index; SBP, systolic blood pressure; sPAP, systolic pulmonary pressure; TPVR, total pulmonary vascular resistance; WU, Wood Units.

RV function also improved significantly from baseline to 48 h, with the TAPSE improvement from 15 ± 4.1 mm to 20.5 ± 4.6 mm [+6.1 ± 4.5 mm (40.7%) mean change, *P* < 0.0001]. There was also a significant reduction in RV strain markers levels at 48 h post-procedure assessment, hs troponin I dropped from 2.4 ± 0.7 ng/mL to 2.1 ± 1.2 [−1.6 ± 7.6 ng/mL (66.7%) mean change, *P* < 0.0001], and NT-proBNP dropped from 5609.7 ± 5805.3 pg/mL to 3319.7 ± 415.9 pg/mL (−3277.4 ± 1667.1 pg/mL [58.4%] mean change, *P* < 0.0001). No significant differences were found in clinical and haemodynamic improvements for patients with HR PE compared to those with IHR PE.

### Safety outcomes

There was one (0.9%) intraprocedural death related to RV failure and four patients (3.6%) experienced sudden cardiac arrest in the catheterization suite with successful resuscitation. In two patients (1.8%) CDMT was complicated by distal subsegmental PA injury and non-massive haemoptysis. In both cases, a 7 Fr thermodilution balloon-tipped catheter (Edwards Lifesciences, USA) was immediately placed and inflated in the segmental artery in proximity to the injured subsegmental artery. The balloon was inflated for ten minutes, with periodic deflations, and angiograms were performed through the distal port of the catheter to determine if the rupture would seal, with final success and no recurrence of haemoptysis. There was no significant drop in haemoglobin concentration in both cases, in the first patient the haemoglobin concentration dropped from the initial 7.4 mmol/L to 6.4 mmol/L, and in the second patient, haemoglobin concentration dropped from the initial 8.0 mmol/L to 6.9 mmol/L after the procedure, respectively.

There were two (1.8%) massive groin haematomas requiring transfusion of two red blood cell packs and qualified as major bleedings within 48 h post-procedure. In both cases, ST was administered as an initial treatment of HR PE. One (0.9%) patient developed an ischaemic stroke with aphasia and left-side paralysis several hours after the CDMT procedure. There were five deaths (4.5%) at 48-h follow-up, three (2.7%) were due to RV failure progression, and 2 (1.8%) were unrelated to the procedure and PE (caused by brain injury due to long-lasting resuscitation). All five patients were HR PE with prior unsuccessful ST with ongoing cardiopulmonary resuscitation (CPR) in whom CDMT was performed as a bailout treatment.

Moreover, 14.5% of patients developed pneumonia or sepsis secondary to pneumonia. There were no additional PE-related deaths at the 30-day follow-up related to PE (overall PE-related 30-day mortality 4.5%), but there was an additional one 0.9% death due to multiorgan failure and two deaths due to disseminated ovarian malignancies (overall all-cause 30-day mortality 8.2%). There were no 90-day all-cause readmission and no further complications. Details are presented in *Tables 4* and *5*.

**Table 4:** Safety and mortality outcomes

Safety outcomes	*N* (%)
*During CDMT procedure*	
PE-related death during the procedure	1 (0.9)
Procedure-related death	0 (0)
Major bleeding during procedure	0 (0)
Pulmonary artery injury during procedure	2 (1.8)
Cardiac tamponade during procedure	0 (0)
Clinical deterioration during the procedure	1 (0.9)
Cardiac arrest during the procedure	4 (3.6)
Sustained ventricular tachycardia during the procedure	1 (0.9)
*48-h follow-up*	
PE-related death within 48 h post procedure	4 (3.6)
All-cause death within 48 h post procedure	6 (5.5)
Major bleeding within 48 h post procedure	2 (1.8)
Pulmonary artery injury within 48 h post procedure	2 (1.8)
Cardiac tamponade within 48 h post procedure	0 (0)
Clinical deterioration within 48 h post procedure	1 (0.9)
Infection (pneumonia/sepsis)	16 (14.5)
Stroke (ischaemic/haemorrhagic)	1 (0.9)
*30-day follow-up*	
PE-related mortality within 30 days post procedure	4 (3.6)
All-cause mortality within 30 days post-procedure	9 (8.2)
PE recurrence	0 (0)
*90-day follow-up*	
PE-related mortality within 90 days post procedure	5 (4.5)
All-cause mortality within 90 days post procedure	9 (8.2)
PE recurrence	0 (0)

Abbreviations: See *Table 1*.

**Table 5:** Detailed characteristics of patients with adverse events

	Patient’s sex, Male/Female; Age, years	Clinical presentation	Indication for CDMT treatment	PE risk category	Adverse event
1	Female; 53	obstructive shock, cardiac arrest with ongoing CPR, intubated	Unsuccessful ST	HR	PE-related death during CDMT
2	Female, 44	Sudden cardiac arrest, witnessed CPR > 1 h during CDMT, intubated, ECMO delivery	Unsuccessful ST	HR	Death within 48 h due to post-traumatic brain injury
3	Male, 50	Sudden cardiac arrest, CPR > 1 h during CDMT, severe COVID-19 infection, ECMO delivery	Unsuccessful ST	HR	multiorgan failure—COVID-19 progression
4	Female, 59	Syncope, obstructive shock, CPR during CDMT ECMO delivery	Unsuccessful ST	HR	PE-related death within 48 h
5	Male, 64	Syncope, obstructive shock,	Unsuccessful ST	HR	PE-related death within 48 h
6	Male, 70	Obstructive shock	Unsuccessful ST	HR	PE-related death within 48 h
7	Female, 84	Presyncope, respiratory failure, ovarian cancer	Relative contraindications to ST	IHR	Death within 30 days due to ovarian cancer
8	Female, 48	Presyncope, respiratory failure, ovarian cancer	Relative contraindications to ST	IHR	Death within 30 days due to ovarian cancer
9	Female, 53	Presyncope, respiratory failure, 24 h after haemorrhagic stroke treatment	Absolute contraindications to ST	HR	Multiorgan failure
10	Male, 44	Obstructive shock	Unsuccessful ST	HR	Major bleeding- access-site haematoma BARC 3a
10	Female, 47	Obstructive shock	Unsuccessful ST	HR	Major bleeding- access-site haematoma BARC 3a
11	Female, 63	Sudden cardiac arrest, witnessed CPR > 1 h during CDMT, intubated, ECMO delivery	Relative contraindications to ST	HR	Pulmonary artery injury during CDMT
12	Male, 48	Obstructive shock	Absolute contraindications to thrombolysis	HR	Pulmonary artery injury during CDMT
13	Female, 60	Presyncope, respiratory failure, cervical cancer	no improvement on low-molecular-weight heparin	IHR	Clinical deterioration during transfer on cath lab table before CDMT
14	Female, 59	Syncope, obstructive shock,	Unsuccessful ST	HR	Sustained ventricular tachycardia when passing through RV outflow tract

Abbreviations: See *Table 1*; BARC, Bleeding Academic Consortium Research; CPR, cardiopulmonary resuscitation; ECMO, extracorporeal membrane oxygenation, ST, systemic thrombolysis.

### Factors associated with postprocedural RV/LV ratio reduction

**Figure 3:**
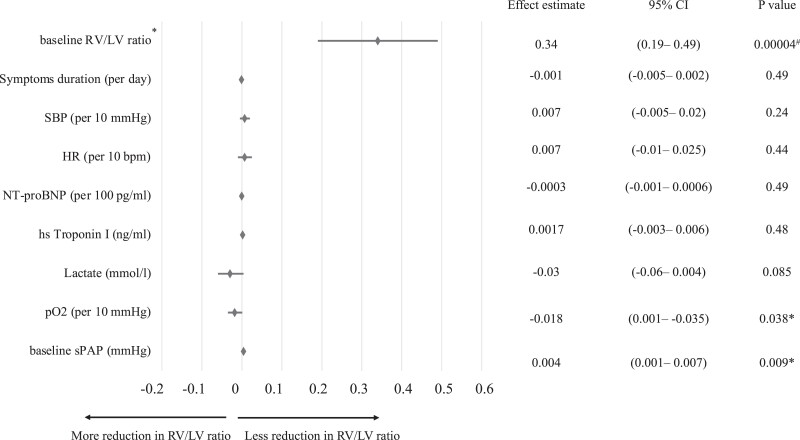
Multiple linear regression model of absolute reduction in RV/LV ratio. *composite RV/LV ratios using either CTPA or transthoracic echocardiography (TTE) measurements, with CTPA prioritized if both were available. ^#^statistically significant.

Multiple linear regression application indicated that higher baseline sPAP was related to a more modest reduction in composite RV/LV ratio following CDMT (*P* = 0.009), while higher initial arterial pO_2_ was associated with a greater reduction of postprocedural RV/LV ratio (*P* = 0.038), respectively (see *Figure 3*).

## Discussion

We observed that CDMT with an 8F Indigo catheter resulted in substantial clot removal and immediate improvement in haemodynamic conditions in PE patients with seemingly low periprocedural risk.

Early mortality in advanced PE is strongly related to haemodynamic compromise due to RV failure.^21^ Increased RV/LV ratio assessed by imaging studies is a reproducible and validated tool for identifying PE patients with an increased risk of early death.^22^ In addition to its prognostic role, the RV/LV ratio change plays a significant role as a marker of therapeutic effectiveness.^21,23^ The mean RV/LV ratio reduction of 0.48 (36%) during 48 h after the procedure is comparable to previous catheter-directed therapy studies including the EXTRACT-PE study (mean RV/LV reduction of 0.43), FLARE trial with the FlowTriever device (mean RV/LV change 0.38), SEATTLE II study (mean RV/LV change 0.42), RESCUE study with the Bashir catheter designed for pharmacomechanical local thrombolysis (mean RV/LV change 0.56) and initial results of mechanical-electric thrombectomy with Magneto 20F device (mean RV/LV change 0.45).^8,21,23–25^ In the recent FLASH registry also with the FlowTriever system, the mean RV/LV ratio drop was 0.25.^10^ What is more, the results of this study indicated a significant change in surrogate markers of RV dysfunction including tachycardia, troponin, NT-proBNP, and lactate levels 48 h after CDMT.

The 10.4 mmHg mean on-table drop in sPAP was like that obtained in the FLASH study (12.8 mmHg), but favourable as compared to the EXTRACT-PE study with the same Indigo aspiration system (4.3 mmHg).^8,10^ We also found that higher baseline sPAP was related to a more modest reduction in RV/LV ratio following CDMT. The mean on-table change in mPAP (−6.1 mmHg) was also similar to that in the FLASH study (−7.6 mmHg).^10^ While studies on the applicability of catheter-directed thrombolysis have also reported significant haemodynamic improvements, these approaches require more time to achieve pulmonary vascular bed decompression in the opposite of CDMT with fast thrombus debulking.^23,26^

In our real-world cohort, significantly more patients (28.2%) had HR PE as compared to previous studies regarding pulmonary CDMT 0.84% to 7.9%, respectively.^8,10,21,27^ This made our population sicker than in previous studies and more frequently excluded from clinical trials. It should be emphasized that our study included patients with polytrauma, malignancies during chemotherapy, and patients with ongoing CPR after the failure of ST in HR PE. Patients with these life-threatening conditions were typically excluded from previous studies. Nonetheless, this study showed a favourable safety profile of CDMT with 3.6% PE-related mortality during the first 48 h after the procedure.

CDMT is increasingly applied in PE treatment for the prevention of patient decompensation and potentially to impact mortality. In this patient cohort, the all-cause 48-h mortality was 5.5% (including 3.6% PE-related). All deaths were HR PE cases in whom CDMT was performed during ongoing CPR after unsuccessful ST. This is significantly lower than reported in a recent meta-analysis of 1517 HR PE patients with an in-hospital of 28.3% despite ST, catheter-directed thrombolysis, or surgical embolectomy.^28^ In the FLASH registry and the EXTRACT-PE study the 48-h mortality was 0.8%, respectively.^8,10^

The all-cause 30-day mortality in this study was 8.1%. One was due to multiorgan failure, and two of those deaths were due to malignancies and were not related to CDMT procedures. None of the previously published studies regarding CDMT devices enrolled patients with malignant neoplasms which significantly impact patients’ survival. A recent meta-analysis of 65 589 patients with PE treated with catheter-based therapy (CDMT or catheter-directed thrombolysis) or ST demonstrated a significantly lower 28–30-day mortality in patients treated with percutaneous techniques vs. ST (7.3% vs. 13.6%).^29^

The important pertinence of CDMT is its utility in patients with high bleeding risk, especially unstable patients with HR PE presentation. In these patients’ ST is absolutely contraindicated, but also catheter-directed local thrombolysis should not be used due to only partial reduction of bleeding risk.^2,26^ Simultaneously, current guidelines indicate surgical embolectomy as a salvage treatment option when other therapies failed.^2,30^ In this study, 34.5% of patients had absolute contraindications for thrombolytic therapy. The major bleedings occurred in 1.8% within 48 h post-procedure and were two access-site bleeds quantified as significant based on haemoglobin drop and necessity of blood products transfusion. Both these patients received ST as an initial treatment of HR PE. Our results are in line with the EXTRACT-PE study assessing the applicability of an 8F Indigo system in 119 IHR PE patients with a 1.7% major bleeding rate which included groin haematoma and vascular injury with the guidewire.^8^ In the present study, two non-massive vessel injuries (1.8%) during CDMT were successfully treated with prolonged balloon inflation and no need for blood product transfusion.

The major bleeding rate in this study was lower than in the ultrasound-assisted catheter-directed thrombolysis SEATTLE II study, where major bleeding occurred in 10% of patients.^23^ However, the overall major bleeding rate reported in recent studies with a lower dose of the locally administered lytic drug (total dose of alteplase ≤ 20 mg) was comparable to our study, 4.0% in the OPTALYSE study, 2.5% in the Standard vs. Ultrasound-assisted Catheter Thrombolysis for Submassive Pulmonary Embolism (SUNSET sPE) study, 2.1% in the CANARY study, and 0.9% in the RESCUE study PMID: 36121244, respectively.^25,26,31,32^ The results of the FLASH registry assessing the safety profile of large-bore CDMT were comparable to ours in terms of major bleeding rate (1.4%) despite FlowSaver blood return use (10.3% of patients).^10^

In the present study, CDMT with an 8F Indigo system was associated with a mean of 9.6% haemoglobin concentration drop due to blood aspiration during the procedure. Despite the lack of a blood return system in the applied 8F Indigo system, no patient needed the transfusion of packed red blood cells because of it. Of note, significant amounts of blood might be aspirated when the catheter is not embedded in the clot, particularly in the hands of inexperienced operators.^33^ Close and serial monitoring of the collection container must be performed to avoid excessive acute blood loss during the continuous aspiration because the system cannot recycle aspirate blood in contrast to the FlowSaver blood return system utilized in large-bore CDMT with FlowTriever since July 2021. The application of FlowSaver reduced the CDMT procedure-related blood loss from about 250 to 100 mL.^10^ In our cohort, the mean periprocedural blood loss was 296 mL. Recently, a new generation of Lightning 12F system (Penumbra Inc, Alameda, CA, USA) was introduced that includes new innovative mechanisms to prevent blood loss, efficiently regulate aspiration and enable aspiration of larger clots.^12,34^ What is more, the next-generation highly torqueable Lightning Flash 16 system (Penumbra Inc, Alameda, CA, USA) with dual clot detection algorithms and no necessity for separator use has just been released in the USA.^35^ The results of the ongoing STRIKE-PE study will show whether Lightning technology ultimately improves short- and long-term clinical outcomes.^36^

It should be emphasized that the CDMT procedure is more technically demanding than catheter-directed thrombolysis and requires familiarity with the PA tree anatomy and expertise to manoeuvre the currently available catheters of varying degrees of stiffness and size through the right heart. The present study focuses on 8F catheters with quite good deliverability into the distal main and lobar PAs. However, the relatively small size of the catheter makes it difficult to remove *en bloc* clots with a bigger diameter. The catheter may also obstruct with clot during the procedure, requiring the application of the separator to clear out the device, and if the separator fails, then the entire device needs to be removed and flushed.^33^ Furthermore, despite the high flexibility of the catheter ensuring manoeuvrability, a passage through the right heart may promote, and/or exacerbate cardiac and/or pulmonary intraprocedural injuries. Moreover, patient selection for CDMT is a work in progress, and next years need to develop trials that clarify the role of this and other catheter technologies in the treatment of severe PE.

### Limitations

Several limitations need to be addressed. First, our study population was relatively small and heterogeneous. Second, this was a single-arm observational study without any comparison group with other treatment modalities or anticoagulation alone. Third, treatment indications were quite heterogeneous, as decisions to perform the CDMT were made by the local PERT. Despite the limited number of study patients, we believe that the consistency of the results validates our observations and helps to improve the understanding of the role of CDMT in IHR and HR PE management. Doubtless, future studies would have to identify, among IHR PE patients, the subgroup of patients with a higher risk of deterioration as potential candidates for urgent CDMT.

## Conclusions

In our real-world single-arm observational registry study of patients treated with the 8F Penumbra system, we observed temporal improvements in RV dysfunction and pulmonary hemodynamics with low observed major bleeding and mortality rates. Currently, experts have no consensus about the optimal method for catheter-directed therapy of PE. Future prospective studies and randomized trials are needed to comparatively evaluate different catheter-directed and pharmacological approaches in PE.

## Data Availability

The data that support the findings of this study are available from the corresponding author upon reasonable request.
